# Memetic Chains for Improving the Local Wireless Sensor Networks Localization in Urban Scenarios †

**DOI:** 10.3390/s21072458

**Published:** 2021-04-02

**Authors:** Paula Verde, Javier Díez-González, Rubén Ferrero-Guillén, Alberto Martínez-Gutiérrez, Hilde Perez

**Affiliations:** Department of Mechanical, Computer and Aerospace Engineering, Universidad de León, 24071 León, Spain; rferrg00@estudiantes.unileon.es (R.F.-G.); amartg22@estudiantes.unileon.es (A.M.-G.); hilde.perez@unileon.es (H.P.)

**Keywords:** Cramèr-Rao Bound, local positioning system, memetic algorithm chains, node location problem, time difference of arrival, variable neighborhood descent

## Abstract

Local Positioning Systems (LPS) have become an active field of research in the last few years. Their application in harsh environments for high-demanded accuracy applications is allowing the development of technological activities such as autonomous navigation, indoor localization, or low-level flights in restricted environments. LPS consists of ad-hoc deployments of sensors which meets the design requirements of each activity. Among LPS, those based on temporal measurements are attracting higher interest due to their trade-off among accuracy, robustness, availability, and costs. The Time Difference of Arrival (TDOA) is extended in the literature for LPS applications and consequently we perform, in this paper, an analysis of the optimal sensor deployment of this architecture for achieving practical results. This is known as the Node Location Problem (NLP) and has been categorized as NP-Hard. Therefore, heuristic solutions such as Genetic Algorithms (GA) or Memetic Algorithms (MA) have been applied in the literature for the NLP. In this paper, we introduce an adaptation of the so-called MA-Solis Wets-Chains (MA-SW-Chains) for its application in the large-scale discrete discontinuous optimization of the NLP in urban scenarios. Our proposed algorithm MA-Variable Neighborhood Descent-Chains (MA-VND-Chains) outperforms the GA and the MA of previous proposals for the NLP, improving the accuracy achieved by 17% and by 10% respectively for the TDOA architecture in the urban scenario introduced.

## 1. Introduction

The precise location of objects in a defined environment is essential for many different activities such as personalized mobile user applications, vehicles navigation, surveillance, or traffic organization. The development of novel cutting-edge technologies is requiring higher localization accuracy and stability for guaranteeing the successful operation of these modern devices.

Traditionally, the localization services have been provided by Global Navigation Satellite Systems (GNSS) which are based on a constellation of satellites in space covering the entire Earth with a reduced number of satellites due to the launching and maintenance costs of each of the satellites of the constellation. Therefore, in some regions, the coverage of these systems is not stable, or the accuracy reached by the system is ineffective for high-demanded applications such as underwater localization [1], autonomous navigation [2], low-level flights [3], or indoor localization [4]. This is due to path signal degradation [5], synchronization effects on system clocks [6], multipath appearance [7], changing in the propagation speed of the radioelectric waves [8], or ionospheric scintillation [9]. Current research on GNSS systems (e.g., GPS) attempts to reduce these limitations through coordinating the satellite signals with cyber-physical systems located in the area of interest for reducing the system uncertainties [10]. Other approaches are considering the design of sensor networks for addressing directly the localization problem with independence of GNSS signals.

As a consequence, Local Positioning Systems (LPS) have emerged as promising systems for addressing the limitations of the GNSS [11]. LPS consist on ad-hoc deployments of sensors which particularly adapt to the characteristics of the environment of application, improving the accuracy and stability of GNSS allowing the precise localization in complex conditions. LPS are classified depending on the physical property measured for determining the target location: Power [12], angle [13], time [14], phase [15], frequency [16], or even combinations of them, thus decreasing the percentage probability of error that each one have individually [17,18].

Among LPS, those based on temporal measurements have the best trade-off among accuracy, stability, robustness, easy-to-implement hardware architectures and costs [19]. Time-Based Positioning architectures are divided considering three main categories depending on the time lapses computed to determine the target location: Time of Arrival (TOA) systems [20], Time Difference of Arrival (TDOA) systems [21], and Asynchronous Time Difference of Arrival (A-TDOA) [22].

TOA systems measure the total time of flight of the positioning signal from an emitter to an architecture receiver. This architecture forces to the synchronization among all the system devices to compute the time measurements. These measurements are then processed to obtain the equation of spheres to solve the target location needing at least 4 receivers to unequivocally determine the target in space.

TDOA systems collect the time measurements resulting from the relative time lapse from the reception of the positioning signal in two different architecture receivers [23]. The measurement of the time without considering the target timestamp allows the unnecessary synchronization of the system devices with the target clock, thus reducing the uncertainties in the position determination generated in the synchronization process. These systems require 5 different receivers to unequivocally determine the target location through the intersection of the hyperboloid equations generated. However, we have demonstrated in [24] that under optimized node distributions, the ambiguity in the position determination through the non-linear intersection of the hyperboloids containing the potential target location, can be solved securely with four sensors.

A-TDOA systems make use of the receive and retransmit strategy of the positioning signal in the target sensor of the signals emitted in the worker sensors of the architecture to a coordinator sensor in which all the time measurements are collected. This methodology allows to get rid of the need to synchronize all the architecture clocks as in the TDOA architecture. This allows the minimum clock uncertainties of the three architectures [25] but increases the signal path used for the target location thus cumulating more noise uncertainties in the transmission.

Consequently, no a priori suitable time-based architecture can be defined in LPS applications [26] and a deep analysis on the environment characteristics must be performed to objectively determine the most appropriate architecture for a particular scenario. In this paper, we analyze the deployment of the synchronous TDOA architecture in a complex urban scenario for their extended use in the literature [27,28] and due to their trade-off among noise and clock uncertainties in LPS applications.

TDOA architecture allows the independence from the emission timestamp of the positioning signal thus reducing the synchronization errors of the TOA architecture and with a reduction in the signal path noise cumulated during the transmission with regards to the A-TDOA architecture which is particularly relevant in complex urban scenarios. Besides, the consideration of all the architecture sensors as coordinator sensors in the TDOA synchronous architecture makes the system more versatile and less dependent on particular sensors of the architecture compromising the system availability in failure cases or partial bad connections with the coordinator sensor of the A-TDOA architecture [19,29].

However, regardless the architecture selected, LPS require an optimization of the node distribution to achieve acceptable results. This problem is known as the Node Location Problem (NLP) [30] and has been considered as NP-Hard [31]. Even, the exclusive consideration of guaranteeing the target coverage by the architecture sensors in the whole coverage design area is already considered as NP-Hard [32]. Consequently, the NLP in localization LPS is addressing a double NP-Hard combinatorial problem [33].

Therefore, heuristic methods have been applied in the literature for solving this complex problem in the field of the Wireless Sensor Networks (WSN). Genetic algorithms (GA) [34,35], the firefly algorithm [36], simulated annealing [37], the bacterial foraging algorithm [38], the grey wolf optimizer [39], the bat algorithm [40], tabu search [41], the elephant herding optimization [42], differential evolution [43], or memetic algorithms (MA) [44] have been applied for achieving practical results for the NLP.

These methodologies require an optimization function for measuring the quality of every node distribution proved in the heuristic algorithms. In the localization field, the Cramèr-Rao Bound (CRB) [45] has been widespread since it is a maximum likelihood estimator which provides the minimum achievable error for a particular sensor distribution regardless the positioning algorithm used for determining the target location. However, the CRB cannot be jointly derived in the entire target coverage area [46] which also promotes the utilization of heuristic techniques for solving the NLP.

In our recent works, we have modeled the CRB through the introduction of the Line-of-Sight (LOS) [47] and Non-Line-of-Sight (NLOS) [48] noise uncertainties introduced in the communications channel during the positioning signal transmission and the clock uncertainties due to the initial-time offset of the synchronization process, the drift of the clocks that compute the time measurement and the resolution of the clock [25]. In this paper, we apply this combined uncertainty model for characterizing the sensor distribution uncertainties.

However, the CRB is a discontinuous function due to the steps generated in the evaluated function among contiguous solutions (i.e., sensor distributions of the space of solutions which minimally differ in a unique spatial coordinate) considering LOS/NLOS propagations of the positioning signal. This discontinuity appears through the difference of the LOS/NLOS path loss exponents which produces notable differences in the signal quality if it faces or saves an obstacle in its propagation. These limitations characterize the NLP in localization as a discontinuous non-derivable combinatorial optimization problem.

In addition, the huge dimensions of the space of solutions due to the necessity of considering a high number of possible sensor distributions for reaching acceptable results for the LPS applications and the high number of decision variables of the algorithm (i.e., the spatial coordinates of each sensor) in large-scale LPS applications also makes the NLP to be considerably challenging.

**Figure 1:**
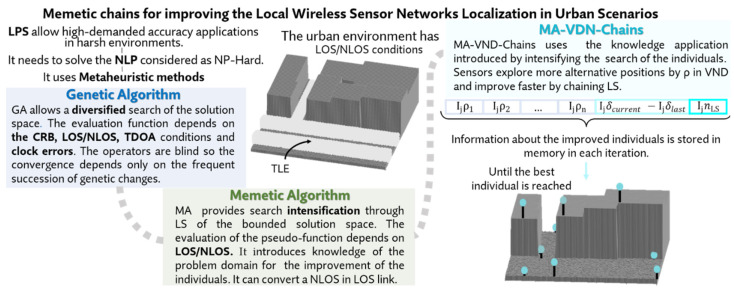
Memetic Chains optimization proposed for the Node Location Problem (NLP).

Therefore, we first proposed a GA [35] for the NLP in LOS environments and we later improved our algorithm through an intelligent employment of the genetic operators during the diversification and intensification phases of the optimization [49]. The dimensions of the space of solutions of the NLP and the consideration of the NLOS paths in the fitness function generating discontinuities in the quality evaluations suggested the introduction of MA (i.e., hybridizations of GA with Local Search (LS) procedures) in both outdoor [44] and urban environments [28]. This memetic strategy for the NLP proved to be promising for analyzing the space of solutions of the NLP which presents numerous local optima complicating the optimization process. Figure 1 shows part of the differences between the GA and MA previously introduced and introduces the new algorithm designed for the NLP in this paper.

Consequently, based on these previous results, in this paper, we introduce a novel MA for the NLP with the consideration of some memory within the LS iterations through the consideration of LS chains [50].

The introduction of these LS chains was proposed in [51] later showing the best results in the competition devoted to Large Scale Global Optimization of the IEEE Congress on Evolutionary Computation (CEC’10) for continuous large-scale optimization problems [52].

However, the characteristics of the NLP with their inherent discontinuities in the fitness evaluations promotes the adaptation of the MA-Solis Wets-Chains (MA-SW-Chains) algorithm introduced in [52] for the discontinuous optimization of the NLP. We adapt this algorithm in this paper through the consideration of a different LS strategy (i.e., Variable Neighborhood Descent (VND [53]) versus the classical Solis Wets algorithm [54] of the MA-SW-Chains algorithm) and the consideration of the movement of the sensor nodes during the LS process for the definition of an intelligent strategy for analyzing the neighborhood of the NLP potential solutions which also differs from our previous strategies implemented in [28,44]. The introduction of the memory chains must also be adapted in this paper to the particularities of the NLP obtaining finally improved results in the solution presented with regards to the previous methodologies proposed for the NLP.

The remainder of the paper is organized as follows: In Section 2 we define the combinatorial NLP together with an analysis of its complexity and state-of-art approaches to this problem, in Section 3 we introduce the scenario of simulations, we present the CRB model for evaluating the fitness of the node distributions analyzed in Section 4, we propose the adaptation of the MA-SW-Chains for the NLP; in Section 5, we show the results obtained through this novel methodology in Section 6 and we conclude the paper in Section 7.

## 2. Node Location Problem in Wireless Sensor Networks

The NLP aims to find the most appropriate location for the sensors in space in order to reduce the system uncertainties [55], maximize the coverage [56], deal with potential sensor failures [29] or reduce the energy consumption of the network [57].

Mathematically, the NLP entails the definition of the three-dimensional Cartesian coordinates of each of the architecture sensors $\left(\langle s_{i}\rangle =\langle x_{i},\;y_{i},\;z_{i}\rangle \right)$. for achieving a particular set of n sensors $\left\{S_{i}=\left(\langle s_{1}\rangle ,\ldots ,\;\langle s_{n}\rangle \right)\right\}$ within the possible combinations of sensors (S) which maximizes the system properties that are enhanced during the optimization process. The definition of the Cartesian coordinates during the optimization must follow a discrete characterization of the LPS coverage space (i.e., Target Location Environment (TLE)) and the space in which the sensor nodes can be located (i.e., Node Location Environment (NLE)) due to the impossibility for dealing with a direct optimization of the NLP without a discretization [35,58].

This makes the NLP to be a combinatorial problem which has been characterized as NP-Hard for guaranteeing the target coverage in wireless sensor networks [32]. In addition, the localization NLP has been also assigned as NP-Hard when a reduction of the system uncertainties is addressed [31]. Therefore, the consideration of the NLP for localization is doubly NP-Hard since the sensor network must provide the least uncertainty in the target location determination but also attaining the stable coverage in the entire TLE.

Consequently, the mathematical model proposed in this paper for the localization NLP is as follows [26]:

(1) $$Maximize\;Z=ff\left(CRB\left(S_{i}\right)\right)$$

Subject to:

(2) $$\;x_{lim_{1}\;}\leq \;x_{i}\;\leq x_{lim_{2}};\;\forall x_{i}\;\in s_{i};\;s_{i}\;\in S_{i};\;s_{i}\notin U$$

(3) $$\;y_{lim_{1}\;}\leq \;y_{i}\;\leq y_{lim_{2}};\;\forall y_{i}\;\in s_{i};\;s_{i}\;\in S_{i};\;s_{i}\notin U$$

(4) $$\;z_{lim_{1}\;}\leq \;z_{i}\;\leq z_{lim_{2}};\;\forall z_{i}\;\in s_{i}\;;s_{i}\;\in S_{i};\;s_{i}\notin U$$

(5) $$Cov_{TLE_{k}\;}\geq n_{min_{TDOA}};\;\forall \;k\in n_{TLE}$$

(6) $$Cov_{TLE_{k}\;}=\;\overset{n}{\underset{i=1}{\sum }}Cov_{TLE_{ki}}$$

(7) $$Cov_{TLE_{ki}\;}=\;\{\begin{array}{c} 1\;\;if\;SNR_{TLE_{ki}}\;\geq SNR_{threshold}\\ 0\;\;\;\;\;\;\;\;\;\;\;otherwise\;\;\;\;\;\;\;\;\;\;\;\;\;\;\;\;\;\;\;\;\;\;\;\; \end{array}$$

where $\;x_{lim_{1}\;}$,$x_{lim_{2}}$,$\;y_{lim_{1}\;}$,$y_{lim_{2}}$,$\;z_{lim_{1}\;}$ and $z_{lim_{2}}\;$ are the lower and upper bounds for the location of the sensors in space respectively; U is the subset of S containing forbidden location for the sensors inside the bounds (i.e., the interior of the buildings in the proposed problem); $Cov_{TLE_{k}\;}$ is the number of sensors that effectively cover a determined *k* TLE analyzed point; $n_{min_{TDOA}}$ the minimum number of sensors which must provide effective coverage for the TDOA architecture for calculating the target location unequivocally that generally can be fixed as 5; $n_{TLE}$ the number of TLE discrete points considered during the optimization process; $Cov_{TLE_{ki}}$ the measurement of the effective coverage of a particular sensor i in a defined TLE point *k*; $SNR_{TLE_{ki}}$ is the Signal-to-Noise (SNR) ratio from the emission of the positioning signal in the target sensor and received in the architecture sensor i and $SNR_{threshold}$ is the value of the SNR from which the positioning signal can be processed in each of the sensor receivers.

The fitness function depends on the CRB which has been proved to be non-jointly derivable for all the TLE points analyzed at the same time [46]. This promotes that derivative methods for finding promising regions of the space of solutions are not recommended. In addition, the consideration of the NLOS paths in the positioning signal transmission makes the problem to be discontinuous. This favors the employment of heuristic methods to address the NLP in the literature [59].

In this paper, we propose a MA with LS Chains for the first time in the NLP in the authors’ best knowledge. We make an adaptation of the so-called MA-SW-Chains which has shown an outstanding performance for Large-Scale Global Optimization problems [60] with continuous fitness evaluations. The characteristics of the NLP described in this section suggest the adaptation of the benefits of this algorithm (e.g., dealing with huge spaces of solutions and the intelligent intensification of promising regions of the space of solutions) to the particularities of this problem (i.e., fitness discontinuity and the introduction of knowledge in the movement of the sensor nodes during the LS procedure).

## 3. The Scenario of Simulations

In this paper, we deploy an optimized TDOA synchronous architecture in an urban scenario. The optimization of the sensor distribution is required for any LPS application in order to achieve practical results. Therefore, we define an NLOS complex urban scenario for testing the capabilities of the novel MA optimization proposed which contains all potential weaknesses for the deployment of the localization architecture (e.g., large buildings which generate difficult connections among the target and the architecture sensors, a harsh environment for the location of the sensors and large area of coverage for the target).

**Figure 2:**
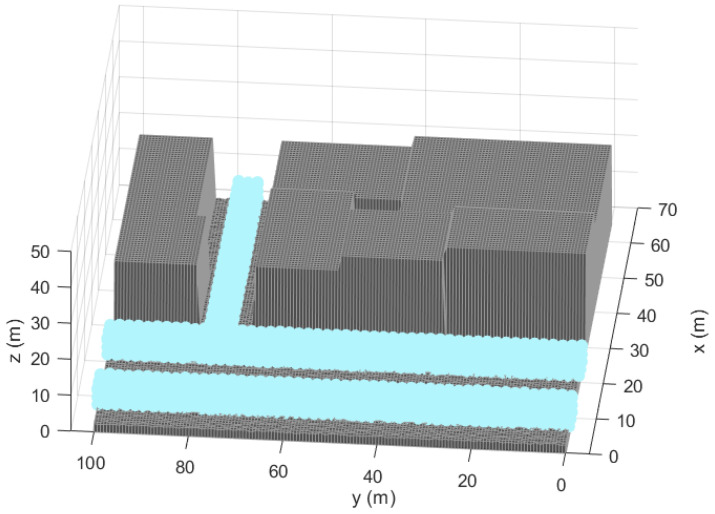
Representation of the 3D urban scenario of simulations with the Target Location Environment (TLE) region in blue showing the coverage of the Local Positioning System (LPS).

We show in Figure 2 the scenario in which we prove the capabilities of the optimization methodology. It comprises a zone of coverage of the LPS, defined as TLE, the zone where the architecture sensors can be located (NLE) and an Obstacle Area (OA) where the sensors and the target cannot be located (i.e., the interior of the buildings) [26]. It supposes a challenging benchmark for the optimization since the LPS is covering two different streets which generates potential difficult links of the positioning signal among the sensors which are particularly covering each of these two regions.

The TLE and the NLE are discretized for preserving the representativity of the space of solutions achieving time-effective results [35]. The TLE is discretized under a spatial resolution of 2 m in the Cartesian coordinates maintaining the representativeness of the scenario since the selection of this value is based on a pre-optimization study from which slightly differences in the statistical parameters involved in the process are achieved considering lower resolutions.

The NLE allows an elevation of the sensors of 2 to 10 m from the base surface (i.e., the ground and the buildings) for avoiding or reducing the multipath effects and other disruptive phenomena on signals produced near the reference surface. The resolution of the NLE varies depending on the dynamic codification of the individuals through a scalation technique that we first introduced in [35]. In this scenario, we make use of binary chains for the codification of the Cartesian coordinates of the individuals (i.e., the decision variables of the algorithm) with lengths of seven, seven and five bits which leads to resolutions of around 0.6 m allowing the access to acceptable solutions for the deployment of the sensor network in LPS applications.

## 4. Cramèr-Rao Bound for the TDOA Synchronous Architecture

The CRB is a maximum likelihood estimator based on the Fisher Information Matrix (FIM) commonly used in the localization field to characterize the quality of a node distribution. The CRB is the common metric for characterizing the quality of a node distribution [61]. It provides the minimum localization error achieved by any algorithm in a defined location given a particular sensor distribution. In [61] a matrix form of the CRB was introduced enabling the characterization of the covariance matrix of the sensor distribution.

In LPS, this uncertainties definition requires a heteroscedastic noise consideration [62] of the architecture at study since the positioning signal varies its path among the target and the different receivers of the architecture. Thus, the heteroscedasticity must be introduced into the covariance matrix of the FIM:

(8) $$J_{mn}={\left(\frac{\partial h\left(TS\right)}{\partial TS_{m}}\right)}^{T}R^{-1}\left(TS\right)\left(\frac{\partial h\left(TS\right)}{\partial TS_{n}}\right)+\frac{1}{2}tr\left(R^{-1}\left(TS\right)\left(\frac{\partial R\left(TS\right)}{\partial TS_{m}}\right)R^{-1}\left(TS\right)\left(\frac{\partial R\left(TS\right)}{\partial TS_{n}}\right)\right)$$

where $J_{mn}$ represents the Fisher Information Matrix (FIM) element (m,n), R is the covariance matrix of the system in which we introduce the uncertainties characterization, h is the vector with the path followed by the positioning signal and consequently with the time measurements of the TDOA architecture at study, TS is the target sensor and *m*, *n*; the spatial coordinates of the target sensor in the reference system employed.

The definition of the covariance matrix must include an actual characterization of the system uncertainties. In this paper, we consider the most extensive consideration of the literature: Noise in LOS [47] and NLOS environments [48] and clock errors [25].

In the TDOA architecture, the time measurements are proved to be correlated [63], thus a complete characterization of the covariance matrix must be accomplished. Therefore, we present the characterization of the h and R for the TDOA architecture:

(9) $$h_{TDOA_{i}}=\|TS-CS_{i}\|-\|TS-CS_{j}\|\;i=1,\ldots ,\;N_{cs}\;\;j=1,\;\ldots \;,\;N_{CS}\;\;\;\;i\neq j$$

(10) $$\begin{array}{cc} {\sigma_{TDOA_{ij}}}^{2}=\frac{c^{2}}{B^{2}\left(\frac{P_{T}}{P_{n}}\right)} & PL\left(d_{0}\right){\left[{\left(\frac{d_{i_{LOS}}}{d_{0}}\right)}_{CS_{i}}+{\left(\frac{d_{i_{NLOS}}}{d_{0}}\right)}_{CS_{i}}^{\frac{n_{NLOS}}{n_{LOS}}}+\left(\frac{d_{j_{LOS}}}{d_{0}}\right)+{\left(\frac{d_{j_{NLOS}}}{d_{0}}\right)}^{\frac{n_{NLOS}}{n_{LOS}}}\right]}^{n_{LOS}}\\ \; & +\frac{1}{l}\sum_{k=1}^{l}\left\{\left|T_{i}-{floor}_{TR}\left(T_{i}+U_{i}-U_{0}+T_{0}\left(\eta_{i}-\eta_{0}\right)+T_{i}\eta_{i}\right)\right|c^{2}\right\}\\ \; & +\frac{1}{l}\sum_{k=1}^{l}\left\{\left|T_{j}-{floor}_{TR}\left(T_{j}+U_{j}-U_{0}+T_{0}\left(\eta_{j}-\eta_{0}\right)+T_{j}\eta_{j}\right)\right|c^{2}\right\} \end{array}$$

where $CS_{i}$ and $CS_{j}$ are the two coordinator sensors involved in the time measurement, $N_{CS}$ is the number of coordinator sensors receiving an effective coverage of the positioning signal emitted from a determined TS location, c the speed of the radioelectric waves, B the signal bandwidth, $P_{T}$ the signal transmission power, $P_{N}$ the mean noise level, $PL\;\left(d_{0}\right)$ the path-loss in the reference distance ($d_{0}$) from which the Log Normal path loss model of [47] is considered, $d_{i_{LOS}},\;d_{i_{NLOS}},\;\;d_{j_{LOS}}\;and\;d_{j_{NLOS}}$ the distances from the TS to the $CS_{i}$ and $CS_{j}$ in LOS and NLOS conditions respectively calculated through the ray tracing algorithm presented in [48]; $n_{LOS}$ and $n_{NLOS}$ the path loss exponents in LOS and NLOS conditions, l the number of iterations of the Monte Carlo simulation performed for estimating the clock uncertainties [25], $T_{i}$ and $T_{j}$ are the total time of flight of the positioning signal from the TS to the $CS_{i}$ and $CS_{j}$ and $U_{i}$, $U_{j}$ and $U_{0}$ and $\eta_{i},\;\eta_{j}$ and $\eta_{0}$ the initial time offset and the drift of the $CS_{i}$, $CS_{j}$ and the reference clock for the system synchronization respectively.

The computation of the inverse of the *FIM* allows the finding of the lower bound for the Root Mean Squared Error (*RMSE*) in a determined target location given the spatial distribution of sensors under analysis:

(11) $$RMSE\;\left(TLE_{i}\right)=\sqrt{trace\left(J^{-1}\right)}$$

## 5. Memetic Algorithm Chains for the Node Location Problem

MAs are a hybridization between a GA and a LS procedure [64]. They are based on the concept of meme which was introduced in the Dawkins theory of transmittable knowledge [65]. A meme is the minimum unit of cultural information with the ability to replicate and evolve affecting the human fitness through reproduction and survival along the generations. Computationally, this idea has inspired the combination of the global optimization strategy of the GA with the intensification of promising regions of the space of solutions for finding the most adapted individuals within this region through a LS method. The LS hybridization favors the introduction of knowledge in the heuristic optimization since it modifies the random intelligent search of the heuristics through the consideration of the particularities of the problem (e.g., in the NLP the movement of the sensor nodes during the Local Search).

The MAs have been applied in many different problems in the literature [66]. In the optimization of the NLP in WSN, MAs have been previously considered for the coverage problem [67,68] and we later extend this procedure to include the positioning accuracy in the localization NLP presented in Section 2 considering both LOS and NLOS links in the signal paths [44].

In this section, we introduce a novel approach to the localization NLP through the consideration of LS chains. This novel optimization methodology presents some modifications from our previous algorithm to improve the results previously obtained. Hereafter, we present the general basis of the LS chains, the pseudo-code of the novel algorithm and the LS procedure in the next subsections.

### 5.1. General Concept of the Local Search Chains

The MA-Chains were presented in [52] in order to develop an algorithm that could be scalable and especially designed for large-scale global optimization. The novelty is the introduction of the intensity involved in the LS using parameters that evaluate the strategies used in each area of the space of solutions. The concept of “*chains*” comes from the number of LS iterations information that the algorithm can combine to improve an individual of the population. It uses an adaptive step, ρ*,* to perform a movement to a better point by weighting the success being achieved in this direction in previous steps of the LS applied to the individual. The improvement of the individual and the information obtained in previous LS iterations is stored for its application in the next LS call.

Therefore, the storage needs the definition of a memory where the LS parameters evolution is stored such as the number of successes or failures, the parameter bias, and the step. In this way, all individuals not involved in any of the GA operators can continue its improvement with this a priori information.

Thus, the algorithm establishes a criterion for choosing individuals, favors those showing more promising skills in the local search area, refines disadvantaged areas and does not penalize exploration (i.e., it tries the avoidance of a premature convergence into a local maximum).

In this paper, we are proposing an adaptation of the MA-SW-Chains to the discrete discontinuous optimization of the NLP. Thus, we introduce some changes in the base algorithm (i.e., MA-SW-Chains) for achieving practical results in this problem through a novel algorithm called MA-Variable Neighborhood Descent-Chains (MA-VND-Chains):

- The introduction of the VND LS in the MA-VND-Chains instead of the SW LS of the MA-SW-Chains for exploring a discrete neighborhood around the individual of the GA selected for the LS.
- The utilization of different knowledge for the selection of the individuals of the LS in the MA-VND-Chains through a different definition of the LS Intensity based not only on the improvement of past LS iterations (i.e., MA-SW-Chains) but also in the information of the size of the neighborhood analyzed around each of the nodes of the NLP and diversity criteria through the number of LS iterations applied previously to the individual selected which is critical to address discontinuous optimization as we previously showed in [44].
- MA-SW-Chains presents a steady-state GA which is not optimal for the NLP since an extensive exploration of the space of solutions is required for achieving practical results. Therefore, we introduce a generational GA in the MA-VND-Chains with high elitism values for preserving the knowledge of the LS Chains on the elitist individuals but guaranteeing a broad exploration of the space of solutions.
- MA-SW-Chains introduces a variable parameter for quantifying the movement of the exploration of the neighborhood region of a solution (ρ) which allows the definition of the distance of exploration of the LS for an individual based on an expected value of improvement in the neighborhood considering previous evaluations. Consequently, only potential solutions distanced ρ from the individual of the LS are explored. Since the NLP requires the addressing of a discrete discontinuous optimization, the methodology of the MA-SW-Chains is invalid since instabilities in the definition of the ρ can appear during the optimization due to changes in the LOS/NLOS paths of the positioning signal between target and nodes. For this reason, the MA-VND-Chains considers a variable neighborhood size around each node in which all the potential discrete solutions around every node are analyzed avoiding the discontinuities in the fitness evaluations. In this sense, the consideration of a promising region for the improvement of the location of each node is considered through an increasing value of the number of points analyzed around the node location.
- MA-VND-Chains considers the evaluation of the promising regions for exploring the neighborhood for each node thus evaluating specifically the potential improvement in the movement of each node during the LS process adapting this algorithm to the NLP and not globally as the MA-SW-Chains.

The following section explains the sequential order used by our algorithm as well as its place in the general framework of the MA-Chains family. Subsequently, its parts are decomposed, its values are detailed, its adaptability to the problem is presented, and its main bullets are discussed.

### 5.2. MA-VND-Chains Pseudo-Code

**Figure 3:**
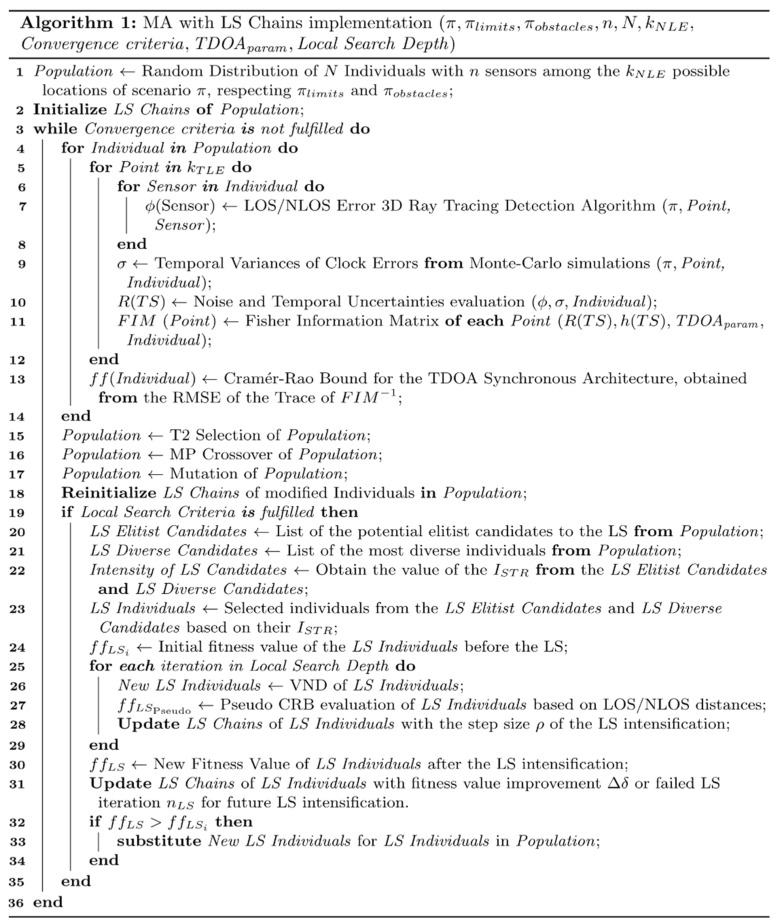
Pseudocode for the memetic algorithms (MA) optimization process followed for the NLP.

The structure of the proposed MA is presented in the following pseudocode:

Figure 3 details the algorithm procedure for optimizing the node distribution. As shown in Figure 3, the optimization process starts with the initialization of a random population and their corresponding LS Chains.

Once created the initial population, the MA takes place. In this algorithm, a standard GA optimization is combined with a LS intensification, with the novelty of the implementation of LS Chains along a degree of memory into the optimization.

Moreover, the introduction of these LS Chains serves as an additional source of knowledge for the problem into the LS, thus improving the optimization procedure. However, these LS Chains are effective when the associated individuals remain unaltered after the LS procedure. Hence, the memory of the intensified individuals that have been modified in the crossover or mutation algorithms is reinitialized after these functions take place.

Furthermore, once the LS criteria is fulfilled, the LS procedure starts by selecting two sets of candidates for the intensification. The first set is composed of elitist individuals, while the second set consists of a selection of the most diverse individuals. Through the evaluation of the intensity $I_{STR}$ of these candidates, a final set of LS individuals is obtained, based on their convenience for the LS intensification.

Therefore, once selected the optimal individuals for the LS, the VND LS takes place. After every iteration of the intensification, the LS chains of the given individuals are updated with the actual step size of the Variable Neighborhood Search (VNS).

Moreover, the LS fitness evaluation consists of a pseudo evaluation of the fitness of the selected individuals. This evaluation is based on the LOS/NLOS distance measurement of the links of the positioning signal among the target and the given nodes, based on the Ray Tracing Algorithm presented in [48]. This pseudo evaluation achieves a lower complexity while obtaining analogous results to those of the main fitness function.

However, once the LS optimization finishes, the intensified individuals are reevaluated under the main fitness value, which considers other relevant factors such as clock errors, temporal uncertainties and geometric factors. Once evaluated, the individuals that show an increase in their fitness value replace their initial candidate before the LS. Nevertheless, the individuals that performed worse than their initial fitness value keep their initial properties.

Nonetheless, the LS Chains of every intensified individual are updated with the corresponding fitness value improvement or failed intensification for future intensity evaluations.

#### 5.2.1. Definition of the Individuals of the LS in the NLP

The MA optimization [69] allows the exploration of adjacent regions in the evolutionary process through the application of the LS to the most different and the elite individuals of the population keeping the balance between diversification and intensification respectively.

The introduction of the most diverse individuals proves essential for preserving the genetic diversity throughout the optimization, thus resulting in a greater degree of exploration in the solution environment. This exploration results vital for avoiding local maximums, especially for highly restrictive scenarios where the existence of considerable NLOS conditions, such as the one presented in this paper, may direct the MA into a non-optimal solution.

Moreover, due to the geometry of these obstacles, minimal changes in the node location may induce significant deviations in the fitness value of the individuals. Therefore, the application of a further LS improves the avoidance of these obstacles, resulting in a considerable enhancement in location accuracy.

The definition of the most diverse individuals is achieved throughout the measurement of dissimilarity among solutions. Therefore, a comparative between every sensor location among individuals is performed, as presented in [44].

On the other hand, the introduction of the elitist individuals into the LS enhances the optimization performance. The intensification of the best suited individuals along the information preserved in their LS Chains achieves a significant improvement in the convergence and final solution of the algorithm.

The balance between intensification and diversification obtained from combining the elite and most diverse individuals, along the introduction of LS Chains and the Intensity parameters achieves a considerable improvement for the overall MA optimization performance.

Moreover, the application of the LS algorithm is regulated throughout the MA convergence with the introduction of the LS Depth. As the algorithm progresses, the convergence thereof begins. Therefore, it increases the frequency of the LS to be executed and the likelihood of obtaining the best individual of its neighborhood. LS Intensity allows the evaluation of the elite individuals to detect if they could be improved through LS or it would be better to introduce a new individual of the population looking for the diversification of the algorithm.

The intensity allows the detection of potentially promising individuals who could be significantly improved when they are running in the LS. In each iteration, the evaluation analyses factors that help the determination of the best candidates for the application of the LS procedure. The calculation of the value of the intensity requires the comparative analysis of how much the fitness improves after LS execution, how often the result of the fitness values is increased through LS, and how much scope for improvement an individual has ahead.

Therefore, in a first step, it is necessary to determine the value of the optimality of each individual involved in the LS before the application of the procedure, and later when the LS execution ends, the algorithm analyzes his fitness back. The results show how much the individuals have improved by the exploration of the LS in comparison with the GA selected individual.

The selection of an individual for the application of the LS looks for the obtention of the best-adapted individual in a bounded region (i.e., the neighborhood of the individual). The more frequenty an individual is selected, the less likelihood to produce an enhancement in its optimal value. Thus, the application of the LS without producing further improvements in the fitness of the individual must penalize this individual allowing a reduction in the possibilities of the selection of the individual in future applications of the LS.

In addition, there also exists some individuals that may be promising but have not been executed by the MA. This is a major factor for determining the need of an individual to be executed in a LS procedure.

(12) $$I_{STR}=C_{1}\times \left(\delta_{current}-\delta_{last}\right)+C_{2}\times \mu_{li}+C_{3}\times \frac{1}{n_{LS}}$$

(13) $$\mu_{li}=\;2\sum^{\;}\frac{\rho }{n}-1$$

where $\delta_{current}$ and $\delta_{ant}$ are the current improvement and the improvement obtained previously, $n_{LS}$ is the number of times that the individual has not improved by LS, $\mu_{li}$ is the factor that determines the need for an individual to be executed in LS, ρ is the step size for VND algorithm, $C_{1},\;C_{2},\;C_{3},$ are experimentally adjusted coefficients to be applied to the reference values for each parameter.

#### 5.2.2. LS Procedure and Chains Memory

The MA introduces problem-specific knowledge for facilitating the improvement of the individuals. As the process of the algorithm converges, the number of individuals that can improved is reduced. MA-Memory introduces knowledge from previous LS iterations to determine the predisposition of an individual to be improved. The reach of the best individual in a bounded region through the LS procedure suggests no future iterations for this individual, thus reducing the computational complexity of the algorithm. Therefore, this individual is not going to be selected for future applications of LS yielding the opportunity to other individuals for improving.

LS is the method that allows the finding of the best-adapted individuals within a bounded region defined. In each execution of the MA, the population is analyzed in terms of elitism, diversification, and intensification, to select a set of potential individuals, that can be improved by the LS. The method selects an individual of the set and executes the VND metaheuristic technique until the maximum number of local iterations permitted is attained. The configuration employed in this search is stored after the whole LS process of an individual. Therefore, this method introduces specific knowledge, heterogeneity in the population, diversity in the evolutionary process, and in addition, accelerates the convergence.

**Figure 4:**
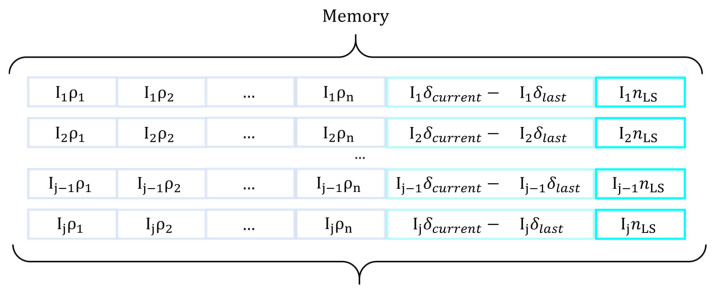
Format of the memory of the MA-Variable Neighborhood Descent-Chains (MA-VND-Chains).

At the outset of the LS, the parameters of the current individual are loaded from the stored memory. This memory provides information about the state of the individual prior to the beginning of the execution. An individual executed for the first time in the LS algorithm has its parameters at the default level. These values are modified as the VND method progresses. The MA-Memory is encoded as shown in Figure 4.

VND obtains an approximate solution to the discrete optimization problem raised to measure the improvement of a sensor by analyzing the adjacent spatial coordinates. The continuous displacement in each visited neighborhood into the best local individual defines the way of the LS optimization. The spatial coordinates of an adjacent sensor node used for localization in LPS must belong to the NLE and not be in the restricted zones. Each time a sensor is displaced, the improved value indicates the k-distance of the next neighborhood. Once the sensor depth search is completed, the value of k-distance is stored in memory. Hence, the k-distance value is stored individually for each of the sensors that composed the individual. The best global fitness of an individual achieved by the VND is reached through one or several LS runs. The memory allows the starting of the VND algorithm in the same state in which the previous LS ended.

The analysis of the changes generated for the MA-VND-Chains algorithm quantifies the improvement introduced with respect to the GA [35] and the MA [28] from our earlier papers. After the LS process, a reevaluation of all the individuals that have been modified through the improvement of the pseudo-function is required. CRB characterizes the quality of a node distribution in the localization field. The individual is modeled according to all the uncertainties associated with the system rather than exclusively with LOS/NLOS conditions as in the pseudo-function.

The individual modified in the MA is replaced when it presents a better fitness than the initial individual. The range for improvement obtained is also quantified and stored in memory. A large improvement increases the chances for an individual to be selected. The individual who does not present an improvement, increases the value of the memory corresponding to the $n_{LS}$ reducing its chances of being executed in future iterations.

#### 5.2.3. Pseudo-Fitness Function

The VND LS pursue the intensification of a given set of individuals in a certain bounded region. In order to obtain the optimal node distribution within this region, the algorithm analyses every possible direction of improvement of the nodes for each individual. Therefore, in each iteration of the algorithm, 26 potential directions of improvement are studied for every node.

The level of complexity of this LS intensification requires a substantial number of fitness value evaluations for each iteration analyzed. Thus, the implementation of a pseudo fitness function for the LS intensification is proposed.

The pseudo function is defined for reducing the complexity of the LS evaluations and it is based on the analysis of the LOS/NLOS links of the positioning signal paths excluding the analysis of the clock and geometric errors with regards to the general fitness function since these two parameters generate practically constant errors in the exploration of the neighborhoods.

(14) $$ff_{LS}=\;\frac{1}{\sum_{k=1}^{T}\sum_{i=1}^{N}\left[d_{i_{LOS}}^{n_{LOS}}+d_{i_{NLOS}}^{n_{NLOS}}\right]+\sum_{k=1}^{T}\sum_{j=1}^{N}\left[d_{j_{LOS}}^{n_{LOS}}+d_{j_{NLOS}}^{n_{NLOS}}\right]\;\;}$$

#### 5.2.4. VND LS

There are different optimization techniques based a neighborhood structure that remains constant during every execution (e.g., Iterated Local Search (ILS) [70]). As the algorithm advances, a rigid structure of the neighborhood ceases part of the effectiveness of the process [71]. In addition, an inefficient search process has a higher computational cost than a more flexible and adapted process to the problem which requires the knowledge of the problem.

The metaheuristic technique VNS solves global optimization problems by changes of neighborhoods. VNS looks for finding in each iteration a better local neighbor for changing the current individual. This ensures that the algorithm will never get a worse result. The method has a deterministic version named highest descent and another variant denominated first descent [72].

In this paper, we apply a Variable Neighborhood Descent algorithm (VND) [73] which explores 26 potential neighborhood movements for each sensor. The VND LS explores all the neighborhood in order to obtain the best-adapted individual in a bounded region (i.e., it is the variant “best improvement” instead of the “steepest descent”). Since all the solutions are scanned, the final result will not change with a different contact order. In each iteration, the analysis of the fitness in the VND algorithm is performed through a pseudo-fitness function in order to compute the progress of the VND procedure.

In the descent-phase, it is difficult to establish the number of neighborhoods to examine, therefore, it is essential to introduce new parameters that allow us to generate heterogeneity in the neighborhood structures and the complementarity of them. Hence, we propose that the structures used in the neighborhoods are dynamic and change depending on the room for improvement achieved in the bounded region above. This method allows for increasing the chances for finding a better global individual by VND compared with a homogeneous structure.

VND begins with the selection of a sensor in order to start the deterministic descent-phase. Every individual is formed of a set of sensors. We randomly select in each iteration the starting sensor for the application of the LS procedure. The randomness favors the diversity of the solution each time the individual is selected by the LS. The determination of the neighborhood structure needs the estimation of a k-distance metric. This parameter modifies the distance that the neighbors occupy concerning the sensor of analysis. When the best neighbor offers a large improvement, the k-distance factor increases in order to reduce the number of iterations needed to obtain the global best location for the sensor.

Depending on the geometry of the sensor distribution, the sensor has a combined LOS/NLOS linkage with the target. In the analysis of a neighborhood, the existence of enhanced individuals which convert an NLOS link to a LOS link causes the value obtained by the pseudo-fitness function of our algorithm to present a large improvement that is awarded with a higher k-distance. Thus, the computational speed required to obtain the best possible individual is lower than with a low and constant k-distance value.

The permutation of the sensors sequence in each epoch arises different LS results, therefore, it is necessary to store the number of times that the individual has not been improved by a LS procedure, $n_{LS},$ and the step size used for each sensor in each individual, as detailed above. The memory will be responsible for storing these values so that the next LS uses the parameters calculated by the previous LS iteration to continue the search for the best individual for its bounded region.

## 6. Results

This section presents the main differences between the previously proposed methodologies (i.e., GA and MA) and the results obtained for the MA-VND-Chains presented in this paper. These results show that the MA-VND-Chains improves the overall performance of the TDOA architecture and reduces the Root Mean Squared Error (RMSE) of the sensor distribution even more than our previous MA [28]. Table 1 shows the parameters used for the positioning signal link and for the architecture sensors characteristics based on real LPS applications.

Figure 2 in Section 3 shows the simulation scenario. The parameters that compose the scenario analyzed are detailed in Table 2.

Improving the GA with the MA increases the complexity of the design but reduces some of its limitations. The GA is inspired by the parameters of biological evolution (i.e., mutation, crossover, and selection) to perform an intelligent search in the space of solutions of the NLP. These genetic operators are blind and do not provide any knowledge about the problem domain.

The MA introduces knowledge of the problem domain to discover new individuals. The LS analyzes the areas discovered in the exploration phase of the GA in search for better individuals through an LS procedure. The balance achieved between diversification and intensification ensures that the MA is more robust to premature convergence. Thus, the RMSE of the TDOA architecture in the covered region by the LPS in the scenario of simulations is lower for the MA than for the GA as shown in Table 4.

The randomness in each iteration favoring the shaking and the acceleration in the descent phase of the MA-VND-Chains algorithm with respect to the MA, allows the faster convergence to the local maximum achieved in the LS and the analysis of more individuals. Table 3 shows the values assumed by these metaheuristic methods.

**Figure 5:**
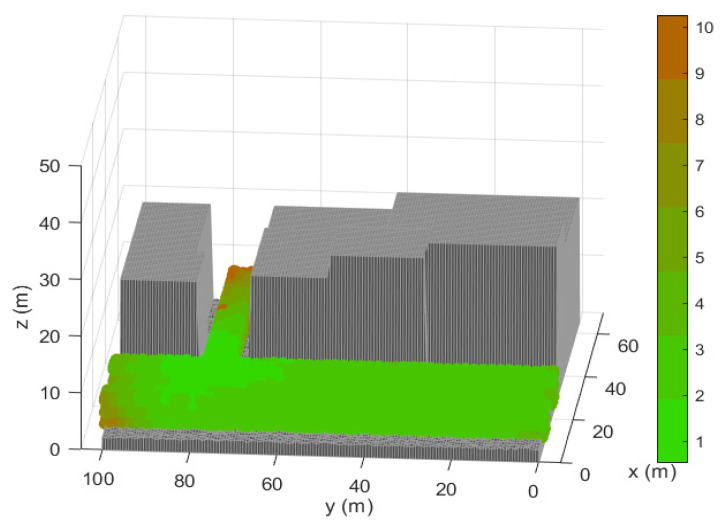
RMSE obtained in the TLE by the best individual reached by the MA-VND-Chains.

The probability of the LS for improving an individual is increased by the intensification parameter which quantifies the need of an individual to be exploited. These measures are reflected in the results obtained by the MA-VND-Chains that reduces the RMSE of the sensor distribution, thus achieving more competitive results. Figure 5 represents the RMSE obtained at each point of the TLE scenario for the MA-VND-Chains distribution.

The simulations have been executed with an Intel Core i7 2.4 GHz CPU and 16 GB of RAM and the algorithm has been coded in the MATLAB platform.

**Figure 6:**
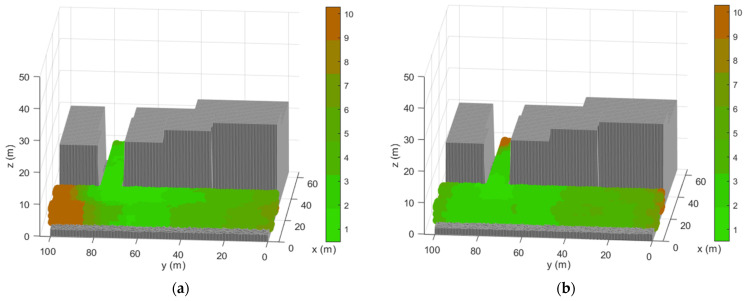
Error obtained in the TLE by the previous techniques implemented: (**a**) RMSE obtained by the GA (**b**) RMSE obtained by the MA.

Figure 6 shows the comparison between the RMSE obtained by the GA, which has not reached an efficient coverage on the scenario, and from the MA, which allows optimizing the sensor distributions by relocating them in adjacent positions to improve coverage in the disadvantaged areas.

The analysis scenario presents mainly three areas with lower accuracy in the coverage zone of the LPS. The areas coincide with the bordering of the space at study. The fitness function tries to maximize the coverage points of the scenario, which benefits sensor locations that are in the center of the scenario for reaching more possible target locations. Due to the exploratory nature of the GA, most of the TLE space is covered with good accuracy. The MA maintains the characteristics of the GA, so it does not increase the number of affected areas but minimizes their size through the reduction of the NLOS links of the architecture.

The MA-VND-Chains achieves even more accurate results than the MA due to the better exploratory technique of the neighborhoods described in this paper. In addition, the attainment of more promising individuals earlier in the optimization process in the MA-VND-Chains algorithm also contributes for reducing the NLOS paths of the positioning signal in the TLE ray-tracing algorithm introduced in [48] thus supposing a major reduction in the time complexity of the optimization since the initial evolutionary steps. This allows the achievement of better results for the MA-VND-Chains algorithm even employing less time in the optimization than in our previous MA. Therefore, these illustrations confirm that the optimization achieved by the MA-VND-Chains allows for a more accurate adjustment of the sensor distribution.

Table 4 shows the percentage of points of the scenario that attain a specific RMSE bound. As it can be seen in the table, the introduction of more knowledge of the problem, produces a reduction in the number of points with large errors in accuracy.

Hence, the results obtained above verify that the proposed technique reduces the areas of lower coverage. The MA-VND-Chains has more points with less error and fewer points with more error, benefiting the accuracy of target positioning. In addition, the results show that it is the technique that has the most points close to the average, improving the overall robustness and the coverage efficiency of the TDOA architecture. Table 5 shows the progressive reduction of the RMSE of the TDOA architecture in the scenario of simulations presented in Section 3 for each of the methodologies proposed (i.e., GA, MA, and MA-VND-Chains).

**Figure 7:**
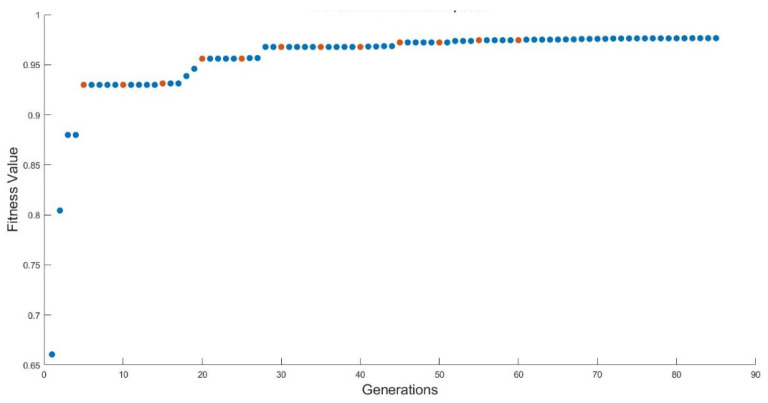
Evolution of the fitness evaluation value of the MA-VND-Chains.

The elitism applied in the metaheuristic methods described guarantees that in each interaction the individual obtained is never worse than the previous one and is closer to the global best distribution. Figure 5 shows the convergence of the MA-VND-Chains methodology that has been investigated and implemented in this article. Figure 7 displays the executions of the VND-Chains LS introduced in this paper in red and the speed at which the algorithm converges for the final best solution.

This is observed in the improvement margin that each iteration undergoes. The method is run using a 9-node distribution. The results reflect the importance of the VND algorithm achieving neighborhood complementarity and reaching better local individuals in each search thanks to the k-distance parameter and the pseudo-function. The MA has more computational complexity and requires more time to arrange the method due to the introduction of knowledge than the GA method. The MA-VND-Chains algorithm does not demand much more complexity to execute than the MA but reduces the execution time.

**Figure 8:**
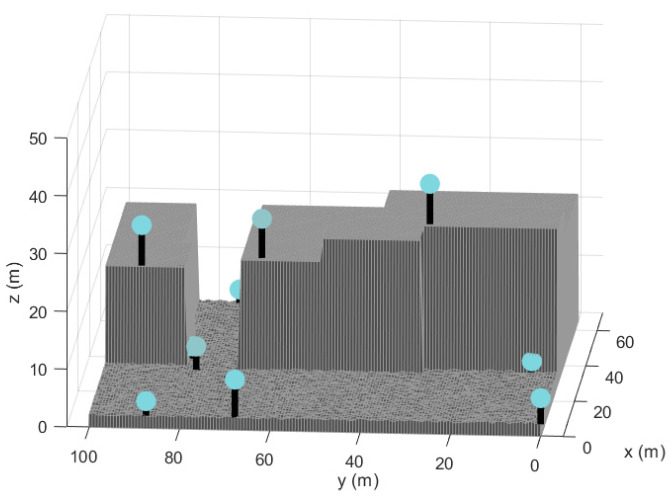
Final configuration obtained by the best individual reached.

Figure 8 represents the optimal nodes configuration, obtained through the MA-VND-Chain optimization introduced in this manuscript. As it is shown in figure, the final distribution of sensors belongs to the NLE, out of the restricted regions.

## 7. Conclusions

Local Positioning Systems (LPS) have attracted considerable research interest during the last few years. They rely on the deployment of sensor networks that particularly adapt to the environment characteristics where they are deployed. This adaptation allows the mitigation of the system uncertainties in harsh environments where the application of Global Navigation Satellite Systems (GNSS) is not recommended for high-demanded accuracy applications such as autonomous navigation, low-level flights, surveillance or indoor localization.

Among LPS, those based on time measurements are the most extended in the literature due to their trade-off among reliability, cost, robustness, accuracy, and easy-to-implement hardware architectures. In this paper, we analyze the Time Difference of Arrival (TDOA) architecture for its application in LPS harsh environments.

However, regardless the architecture used, LPS require an optimization of the sensor distribution in space in order to achieve competitive results. This supposes the solution of the Node Location Problem (NLP) which has been assigned as NP-Hard. Therefore, the employment of metaheuristics for this problem has been widespread in the literature. In this paper, we first introduce Local Search (LS) chains for optimizing the LS procedure of a Memetic Algorithm (MA) for the NLP. This enables the reduction of the algorithm complexity and a more intelligent exploration of the huge space of solutions of the localization NLP.

For this purpose, we perform an adaptation of the so-called MA-SW-Chains algorithm for the NLP. This implies the transformation of this continuous global optimization methodology to discrete discontinuous optimization. This supposes the application of the Variable Neighborhood Descent (VND) LS instead of the Solis Wets LS of the MA-SW-Chains and the selection of the individuals of the LS based on diversity and elitist criteria for analyzing potential unfavored regions of the space of solutions in the evolutionary optimization of the NLP.

In addition, the new algorithm introduced in this paper, MA-VND-Chains, utilizes a pseudo-function in the LS for reducing the complexity of the fitness evaluation based on the slight variations in the clock and geometric uncertainties of the NLP inside a bounded region analyzed (i.e., neighborhood). This entails the definition of a pseudo-function that exclusively considers the LOS/NLOS paths of the positioning signal among target and architecture sensors in the neighborhood LS. Consequently, the acceleration of the LS calculations enables incremental values of the LS depth and LS frequency for attaining improved results in the NLP.

The consideration of the memory chains through the MA-VND-Chains algorithm in this paper enables the introduction of the setting of the hyperparameters of the LS based on previous iterations of an individual. This allows both an enhanced search in the neighborhoods and the adequate selection of the best candidates to apply the LS considering a parameter called intensity which is analyzed based on the improvement of the fitness of an individual in previous LS iterations, the analysis of the neighboring solutions examined in the last LS iteration and the frequency of selection of the individual for LS conserving the diversity as an important factor to determine the LS candidates.

This novel methodology applied in an LPS urban scenario outperforms our previous optimization algorithms proposed for the NLP by 17% with regards to GA and by 11% with regards to MA in terms of accuracy of the TDOA architecture. This improvement shows the validity of this technique for the NLP optimization and presents the MA-VND-Chains methodology as an interesting algorithm for discrete discontinuous optimization thus fulfilling the main objectives of this paper.

## Figures and Tables

**Table 1:** Values used for the configuration of the distributions [74,75,76].

	Parameter	Magnitude	Measure Unit
Link	Frequency of emission	5.465	GHz
	Bandwidth	100	MHz
	Transmission power	1	W
	Mean noise power	−94	dBm
	Line-of-Sight (LOS) path loss exponent	2.1 ^1^	
	Non-LOS (NLOS) path loss exponent	4.1 ^2^	
Sensor	Receptor sensibility	−90	dBm
	Clock frequency	1	GHz
	Frequency-drift	U{−15, 15}	ppm
	Time-frequency product	1	

^1,2^ Path-loss exponents used in the Log-Normal model.

**Table 2:** Parameters for the urban scenario configuration.

	Parameter	Magnitude
Points analyzed	TLE	1471
	NLE	24,000
	RMSE_*ref*_	300 m

**Table 3:** Values used by the Genetic Algorithm (GA), Memetic Algorithm (MA) and MA-Variable Neighborhood Descent-Chains (MA-VND-Chains) [49].

	Parameter	Magnitude
Population	Individuals	80
	Elitism	13%
Operators	Selection	Tournament 2
	Crossover	Single-point
	Mutation	3%
LS	Depth	5
	Individuals	15%
	Algorithm	VND
Intensity	Threshold	1
	ρ	≥0.25 m

**Table 4:** The percentage of points of the TLE for the proposed techniques with different error bounds.

Interval for RMSE Value (m)	GA (%)	MA (%)	MA-VND-Chains (%)
Less than 2.5	35.64	26.76	**47.79**
Less than 5	76.43	84.96	**89.26**
More than 5	23.57	15.04	**10.74**

**Table 5:** Mean of the Root Mean Square Error (RMSE) in the TLE analyzed points in the scenario of simulations.

Number of Nodes	GA [35] (m)	MA [28] (m)	MA-VND-Chains (m)
9	4.30	4.02	3.57

## Data Availability

Not applicable.
